# Vickers hardness prediction from machine learning methods

**DOI:** 10.1038/s41598-022-26729-3

**Published:** 2022-12-28

**Authors:** Viviana Dovale-Farelo, Pedram Tavadze, Logan Lang, Alejandro Bautista-Hernandez, Aldo H. Romero

**Affiliations:** 1 Department of Physics, West Virginia University, Morgantown, WV 26506 USA; 2 Facultad de Ingeniería, Benemérita Universidad Autónoma de Puebla, Edificio ING2, Ciudad Universitaria, 72570 Puebla, Mexico

**Keywords:** Condensed-matter physics, Mechanical engineering, Structure of solids and liquids

## Abstract

The search for new superhard materials is of great interest for extreme industrial applications. However, the theoretical prediction of hardness is still a challenge for the scientific community, given the difficulty of modeling plastic behavior of solids. Different hardness models have been proposed over the years. Still, they are either too complicated to use, inaccurate when extrapolating to a wide variety of solids or require coding knowledge. In this investigation, we built a successful machine learning model that implements Gradient Boosting Regressor (GBR) to predict hardness and uses the mechanical properties of a solid (bulk modulus, shear modulus, Young’s modulus, and Poisson’s ratio) as input variables. The model was trained with an experimental Vickers hardness database of 143 materials, assuring various kinds of compounds. The input properties were calculated from the theoretical elastic tensor. The Materials Project’s database was explored to search for new superhard materials, and our results are in good agreement with the experimental data available. Other alternative models to compute hardness from mechanical properties are also discussed in this work. Our results are available in a free-access easy to use online application to be further used in future studies of new materials at www.hardnesscalculator.com.

## Introduction

Hardness is a measure of the resistance of a material to localized plastic deformation. Over the years, several hardness-testing techniques (like Brinell, Vickers, Knoop and Rockwell) have been developed, and each one has its own scale. However, the basic principle to measure hardness is to force an indenter into the surface to be tested under controlled load conditions. The larger the indentation, the softer the material. The depth and size of the indentation are then converted into a hardness number. In this work we will focus on Vickers hardness, which is one of the most popular techniques given that it is experimentally easy to calculate and can be used for all materials regardless of hardness. Vickers hardness test uses a very small diamond indenter with a pyramidal geometry that has an angle of 136$^\circ$ between the plane faces of the indenter tip. The Vickers hardness measurement is determined by the following ratio:

1 $$\begin{aligned} H_v = 1.854 F / d^2, \end{aligned}$$

where *F* is the applied force (kgf) and *d* is the average length of the diagonal left by the indenter (mm).

The search for new materials with superior hardness has generated considerable interest in the scientific community for many years^1–3^. These materials are needed in extreme industrial applications, such as hard cutting tools, abrasion, and wear-resistant coatings. Traditionally, diamond, titanium nitride, and cubic boron nitride (c-BN) are the preferred materials for these applications. However, they have limitations due to the difference in the chemical bonding character and chemical reactivity. For example, diamond reacts with iron, and the synthesis process of the first two materials requires high-pressure and high-temperature conditions making them costly^4^.

First principle methods have demonstrated to be viable for predicting many physical properties of materials. Among many existing techniques, density functional theory (DFT) stands out for its practical and helpful approach to solving condensed matter systems. DFT has become a primary tool for calculating crystal structures and elastic properties of a wide range of materials with remarkable success when comparing the results to experiment^5^. However, predicting hardness from ab initio calculations is not a trivial task. Hardness is a measure of the resistance of a solid to plastic deformation^6^. Despite its success in calculating elastic properties, DFT cannot predict a solid’s plastic behavior directly.

In recent years, correlations between the elastic properties and the plastic behavior of materials have been established to evaluate the hardness from a theoretical approach^4,7,8^. A hard material will exhibit a slight indentation. The observed shape can be correlated to the elastic response a hard material should have: be incompressible (high bulk modulus), not deform in a direction different from the applied load (high shear modulus), and not distort plastically (strong directional bonds that prevent the creation and motion of dislocations)^4^. The Poisson’s ratio relates the bulk modulus and the shear modulus. A high shear modulus requires a high bulk modulus and a small Poisson’s ratio. A low value for the Poisson’s ratio results from directional bonds in the crystal^4,8^. For example, the Poisson’s ratio for diamond is 0.07, 0.1 for a typical covalent material, and 0.3 for an ionic one^8^. On the other hand, the resistance of a material to plastic deformation depends on the chemical environment of the crystal; a material with short covalent bonds will minimize the activation and mobility of dislocations enhancing the hardness. Thus, covalent materials are generally harder than ionic or metallic^4^. Given the complexity of the problem, there is no universal method that predicts hardness accurately from previously known properties of a material.

With these ideas in mind, several semi-empirical relations between hardness and elastic properties of materials have been proposed over the years^7,9–12^. Usually, these correlations reasonably agree with the experiment for a specific set of materials, but they would not hold when extrapolating to a wide variety of solids.

**Figure 1:**
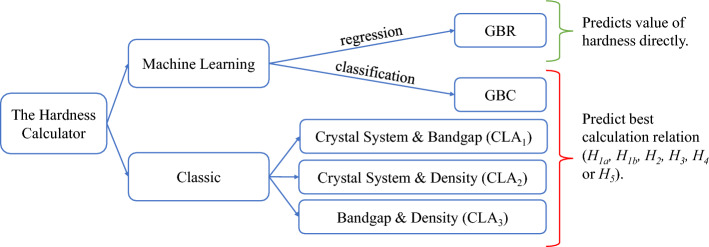
Conceptual diagram of the hardness calculator.

In this investigation, we proposed various models to compute hardness using the mechanical properties of a solid. The mechanical properties (bulk modulus, shear modulus, Young’s modulus, and Poisson’s ratio) were obtained from the theoretical elastic tensor. As shown in Fig. 1, we used two approaches: classic and machine learning (ML).

In the classic approach we studied the six different macroscopic relations for hardness nicely presented by Ivanovskii in Ref.^13^, listed in Eqs. (2)–(7), with a database of more than 140 materials. These relations depend solely on mechanical properties. We calculated the Vickers hardness ($H_v$) using the six relations and compared the results with the experiment to evaluate which method is more suitable for each material kind. We observed the correlation between the six different hardness relations and some physical properties of solids (crystal system, bandgap, and density). From this approach, we developed *The Classic Calculator*, a selection model of the best relation to compute hardness based on simple properties of a solid.

Given the exponential growth in computing power and the development of highly efficient algorithms, machine learning is used today to solve numerous kinds of problems^14^. In the second part of this study, we built a successful machine learning regression model (GBR) to predict the value of hardness directly using the mechanical properties of a solid as input variables. This model demonstrated the highest predicting power among all proposed models in this work. However, given that many scientists use machine learning with hesitation, we also created a classification ML model (GBC) that predicts the best relation to compute hardness with the same data and input variables. This method allows users to select the best relation to compute hardness using the robustness of modern ML algorithms without losing track of the physics behind the calculation. Both ML models, GBR and GBC, are referred to as *The Machine Learning Calculator* in this work.

Both, classic and ML schemes, are discussed, compared to each other, and used successfully to predict new hard and superhard materials. In general, *The Machine Learning Calculator* has proven to be more accurate than *The Classic Calculator*. However, both schemes have demonstrated superior predicting power. The most accurate model was proven to be the machine learning GBR, followed by GBC, and the classic model that uses crystal system and density simultaneously.

This investigation aims to provide valuable tools for the theoretical prediction of hardness. *The Hardness Calculator*, which includes classic and ML predictors, is presented in a free access online application for users to discriminate between the different available results. We believe *The Hardness Calculator* stands out among other methods proposed in the past because: (1) it can be used for a wide variety of solids, (2) it’s easy to use, (3) it is available for everyone as a free-access website that does not require any coding knowledge, (4) and it provides different hardness models simultaneously. Even though GBR is the recommended model in this work, users have the option to consider GBC or any of the classic calculators instead.

## Methods

For most of the database, the elastic tensor was extracted from the Materials Project’s database^15^, while for a few materials (18), it was calculated using first principles. The latter materials were added to the database to ensure a wide variety of materials for the study. The subsequent elastic properties: bulk modulus (*B*), shear modulus (*G*), Young’s modulus (*Y*), and Poisson’s ratio ($\nu$) were calculated using the MechElastic package^16^. The detailed database used in this investigation, including the experimental hardness and the mechanical properties, is presented in the supplemental information.

The first-principles calculations were performed within the framework of DFT^17^. The exchange and correlation effects were treated using the Generalized Gradient Approximation (GGA) with the parameterization of Perdew–Burke–Ernzerhof (PBE)^18^. The valence electrons wave functions were described by the projector augmented-wave method (PAW)^19^. The cutoff energy and the gamma-centered k-point mesh^20^ were converged in each case to assure a maximum error of 1 meV/atom. The self-consistent electronic loop was set to a maximum total energy difference of $10^{-6}$ eV. The calculations were performed using the Vienna Ab initio Simulation Package (VASP)^21–24^.

### Semi-empirical relations for hardness

For each material, the Vickers hardness was estimated using the following six different semi-empirical relations:

2 $$\begin{aligned}{} & {} H_{1a} = 0.1475 \times G \rightarrow Ref. {^{7}}\end{aligned}$$

3 $$\begin{aligned}{} & {} H_{1b} = 0.0607 \times Y \rightarrow Ref.{^{7}} \end{aligned}$$

4 $$\begin{aligned}{} & {} H_{2} = 0.1769 \times G - 2.899 \rightarrow Ref.{^{9}} \end{aligned}$$

5 $$\begin{aligned}{} & {} H_{3} = 0.0635 \times Y \rightarrow Ref.{^{10}} \end{aligned}$$

6 $$\begin{aligned}{} & {} H_{4} = \frac{(1-2 \nu ) B}{6 \ (1+ \nu )} \rightarrow Ref.{^{11}} \end{aligned}$$

7 $$\begin{aligned}{} & {} H_{5} = 2(k^2 G)^{0.585}-3; \ k=G/B \rightarrow Ref.{^{12}}. \end{aligned}$$

Each result was compared to the experimental value in order to determine the absolute error in each calculation. The absolute error was defined as the absolute value of the difference between the experimental ($H_{exp}$) and the predicted ($H_{pred}$) Vickers hardness as shown in the following equation.

8 $$\begin{aligned} \text {Absolute Error} = |H_{exp} - H_{pred} |. \end{aligned}$$

For example, diamond is known as the hardest bulk material with an experimental Vickers hardness of 96 GPa. From the elastic tensor provided in the Materials Project’s database (mp-66), we calculated its theoretical bulk modulus ($B = 435$ GPa), shear modulus ($G = 521$ GPa), Young’s modulus ($Y = 1117$ GPa), and Poisson’s ratio ($\nu = 0.07$). Using these results, it’s possible to estimate the hardness of diamond using the six relations listed in Eqs. (2)–(7) as follows: $H_{1a} = 76.8$ GPa, $H_{1b} = 67.8$ GPa, $H_{2} = 89.3$ GPa, $H_{3} = 70.9$ GPa, $H_{4} = 58.3$ GPa and $H_{5} = 93.0$ GPa. As observed, some relations work better than others. The absolute error (Eq. 8) reveals the accuracy of each relation when predicting hardness of a given material. For the case of diamond, the best relation to estimate hardness is $H_{5}$ because it exhibits the lowest absolute error (3.0 GPa).

To determine which hardness calculation method is more suitable for each type of material, they were classified by crystal system, electronic bandgap ($\Delta E$), and density ($\rho$). According to the bandgap, materials were defined as insulators ($\Delta E > 2 eV$), semiconductors ($\Delta E < 2 eV$) and metals ($\Delta E =0$). Additionally, the compounds were arranged by low ($\rho <4$ g/cm$^3$), medium (4 g/cm$^3 \le \rho \le$ 9 g/cm$^3$) and high density ($\rho>$ 9 g/cm$^3$). Each of these models was analyzed and compared to each other to establish which is more effective in minimizing the mean absolute error (MAE) in the hardness calculation. The MAE is defined in Equation 9, where N is the number of samples.

9 $$\begin{aligned} \text {MAE} = \frac{1}{N} \sum _{N} |{H_{exp}} - {H_{pred}} |. \end{aligned}$$

Further correlations, including two variables simultaneously (*Crystal System + Bandgap*, *Crystal System + Density*, and *Bandgap + Density*), were also studied.

### Machine learning

To find a methodology that predicts the hardness based on different elastic properties, we have used diverse supervised learners, where hardness is the expected output, and the user needs to provide the mechanical properties of a solid (*B*, *G*, *Y*, $\nu$) as input variables. There are two types of supervised learning techniques: classification and regression. In this study, the classification algorithms target the best hardness calculation relation ($H_{1a}$, $H_{1b}$, $H_{2}$, $H_{3}$, $H_{4}$, or $H_{5}$), while the regression algorithms aim to predict the value of hardness directly. Therefore, to generate and compare different algorithms, the created experimental database of 143 materials was split into train and test sets, where the train set has 80% of the data, and the test set the remaining 20%. This approach is essential to have an out-of-sample accuracy.

#### Classification

Supervised learning classification algorithms such as K-Nearest Neighbors (KNN), Decision Trees (DT), Logistic Regression (LR), Support Vector Machines (SVM), Random Forest (RF), AdaBoost (ADA), and Gradient Boosting Classifier (GBC) were used to generate algorithms capable of predicting the best hardness calculation relation given the mechanical properties of a material (*B*, *G*, *Y*, and $\nu$) as an input^25^.

KNN finds the *k* closest training examples (*k* is the number of nearest neighbors) and assigns the new object with the most common class among its *k* nearest neighbors. DT is an algorithm that splits the data according to certain parameters, in this case the mechanical properties. LR works with the probability of an object belonging to a certain class. SVM is an algorithm that classifies cases by finding a separator or a boundary. RF is built by a multitude of decision trees, and the output is the class selected by most trees. ADA is built by a multitude of weak learners each one with a different weight, and the output is the class that gets the most points in the weighted sum. Gradient boosting (GBC for classification tasks) is an ensemble of decision trees that are built subsequently based on the errors of the previous tree. All trees have equal saying in the final output.

The KNN algorithm was optimized for a k-parameter of three neighbors. The DT classifier was defined for a maximum tree depth of three. The inverse of regularization strength for LR was set to 0.01, and the solver liblinear was used given it is the best for small datasets. The SVM was trained with the Radial Basis Function kernel. The RF was built with a maximum tree depth of two and a random seed of zero. The ADA classifier was set with a maximum number of estimators equal to 100 and a zero random seed. The GBC was parameterized with 100 estimators, a maximum depth of the individual regression estimators of 1, a learning rate of 0.6, and a random seed of zero. The rest of the parameters have default values in all cases.

The different classifiers were compared using out-of-sample accuracy and Jaccard index. These metrics are defined as follows:

10 $$\begin{aligned}{} & {} \text {Accuracy} = \frac{1}{N} \sum _{N} 1(\hat{y_i} = {y_i}), \end{aligned}$$

11 $$\begin{aligned}{} & {} \text {Jaccard index} = \frac{y \cap {\hat{y}}}{y \cup {\hat{y}}}, \end{aligned}$$

where N is again the number of samples, ${\hat{y}}$ are the predicted labels, and *y* are the actual labels. The MAE was also computed in each case.

#### Regression

Gradient boosting can be used in regression and classification tasks. To predict the hardness directly, the Gradient Boosting Regressor (GBR) was implemented^25^. GBR is a supervised learning regression technique that creates a prediction model with the same input variables used before (B, G, Y, $\nu$). The algorithm was only parameterized with a random seed of zero. All the other parameters have default values. The MAE was also computed to measure the accuracy of the model.

## Results and discussion

### Comparing different relations of hardness

We started by defining the best hardness calculation relation based on the crystal system. As observed in Table 1, for the 143 structures considered in this study, relation $H_{1a}$ is the most accurate, with an MAE of 3.3 GPa. This relation is also the preferred one for cubic structures. Nevertheless, some crystal systems work better with other approximations. The hexagonal, monoclinic, and tetragonal groups prefer the $H_{4}$ relation, while the orthorhombic and trigonal types minimize their MAE by using $H_{2}$. The triclinic group works better with the $H_{5}$ relation. Calculating the hardness with the selected relation for each crystal type reduces the general MAE from 3.3 to 3.0 GPa.

**Table 1:** Hardness MAE (GPa) for various materials classified by crystal system, using six different semi-empirical relations. *Materials* specifies the number of compounds considered for each crystal system. The *Min Error* value corresponds to the method that minimizes the error in each case.

	Cubic	Hexagonal	Monoclinic	Orthorhombic	Tetragonal	Triclinic	Trigonal	General
Materials	55	18	8	27	15	5	15	143
Error $H_{1a}$	2.9	3.6	3.3	4.6	2.2	1.4	4.3	3.3
Error $H_{1b}$	3.3	3.4	3.5	5.0	2.4	1.5	5.0	3.7
Error $H_{2}$	3.0	5.2	2.9	4.5	2.9	1.9	3.2	3.5
Error $H_{3}$	3.3	4.1	3.9	5.2	2.7	1.6	4.9	3.9
Error $H_{4}$	4.3	2.8	2.3	5.4	1.6	1.3	6.4	4.0
Error $H_{5}$	3.1	5.2	3.9	5.5	3.7	1.2	5.0	4.1
Min Error	2.9	2.8	2.3	4.5	1.6	1.2	3.2	3.0

As observed, systems with all lattice parameters equal to each other (cubic and trigonal) work successfully with relations of hardness that depend solely on the shear modulus ($H_{1a}$ and $H_{2}$ respectively). On the other hand, systems with all angles equal to 90$^{\circ }$ (cubic, orthorhombic and tetragonal) do not display such a clear trend. While cubic and orthorhombic systems also work better with the shear modulus ($H_{1a}$ and $H_{2}$), tetragonal systems prefer a combination of the bulk modulus and Poisson’s ratio ($H_{4}$), and the shear modulus appears as the second-best option ($H_{1a}$). Nevertheless, the latter results suggest that, in general, for high-symmetry systems, the shear modulus is a good descriptor of hardness. Perhaps, it is simple to capture the overall rigidity of a solid in a single parameter if the system is highly-symmetric.

On the other hand, systems with two of their lattice parameters equal to each other and well-defined angles (hexagonal and tetragonal) exhibit an inclination toward a combination of the bulk modulus and Poisson’s ratio ($H_{4}$). Notably, having an expression that depends simultaneously on these two parameters provides significant flexibility in describing the rigidity of a solid in these cases.

Finally, low-symmetry systems, with all lattice parameters different from each other and at least one angle different from 90$^{\circ }$ (monoclinic and triclinic), exhibit a preference for the combination of the bulk modulus with another property. Monoclinic structures work better with the combination of bulk modulus and Poisson’s ratio ($H_{4}$), while triclinic structures prefer the combination of bulk and shear modulus ($H_{5}$).

**Table 2:** Hardness MAE (GPa) for various materials classified by bandgap (Insulators, Semiconductors and Metals), using six different semi-empirical relations. *Materials* specifies the number of compounds considered in each classification. The *Min Error* value corresponds to the method that minimizes the error in each case.

	Insulator	Semiconductor	Metal	General
Materials	22	53	68	143
Error $H_{1a}$	3.9	3.3	3.1	3.3
Error $H_{1b}$	4.9	3.6	3.3	3.7
Error $H_{2}$	2.6	3.4	3.9	3.5
Error $H_{3}$	4.7	3.7	3.7	3.9
Error $H_{4}$	7.5	4.1	2.8	4.0
Error $H_{5}$	4.5	3.8	4.2	4.1
Min error	2.6	3.3	2.8	3.0

Similar to the previous discussion, additional analyses were performed but now considering different electronic bandgaps (insulators, semiconductors, and metals) and density (low, medium, and high) as criteria to distinguish the elastic response. The general MAE was 3.0 GPa and 2.6 GPa, respectively.

Table 2 displays the details for the bandgap classification. The best approach for insulators is $H_{2}$, while for semiconductors is $H_{1a}$, and for metals $H_{4}$. These results indicate that for insulators and semiconductors, the shear modulus is a better descriptor of hardness, while metallic systems work better with a combination of bulk modulus and Poisson’s ratio. The latter result suggests that the shear modulus can capture a solid’s overall rigidity when it is composed of strong directional atomic bonds.

**Table 3:** Hardness MAE (GPa) for various materials classified by density (High, Medium and Low), using six different semi-empirical relations. *Materials* specifies the number of compounds considered in each classification. The *Min Error* value corresponds to the method that minimizes the error in each case.

	Low	Medium	High	General
Materials	26	94	23	143
Error $H_{1a}$	5.3	2.9	2.8	3.3
Error $H_{1b}$	6.7	3.0	2.9	3.7
Error $H_{2}$	3.5	3.4	4.1	3.5
Error $H_{3}$	6.1	3.4	3.4	3.9
Error $H_{4}$	11.4	2.3	2.7	4.0
Error $H_{5}$	4.6	4.1	3.5	4.1
Error $H_{5}$	4.6	4.1	3.5	4.1
Min Error	3.5	2.3	2.7	2.6

Table 3 presents the details for the density analysis. Materials with a low density behave better with the $H_{2}$ approximation, while materials with medium or high-density incline for $H_{4}$. This observation aligns with the previous findings, given that low-density materials usually have strong directional bonds and small packing factors, while high-density materials have metallic bonds and close-packed crystal structures.

**Table 4:** Comparison of the hardness MAE (GPa) and standard deviation $\sigma$ (GPa) for various classification methods. Accuracy was calculated with respect to the best possible result.

Classification method	MAE	$\sigma$	Accuracy
Best possible result	1.0	1.2	100%
Crystal system	3.0	3.2	23%
Bandgap	3.0	3.7	24%
Density	2.6	2.7	31%
Crystal system + bandgap (CLA$_1$)	2.3	2.7	34%
Crystal system + density (CLA$_2$)	2.2	2.2	34%
Bandgap + density (CLA$_3$)	2.5	2.9	36%

A similar exercise including two variables simultaneously was executed to minimize the absolute error. Table 4 presents the results for the different single and combined methods. The first row presents the best possible result; when the hardness of each material is calculated with the relation ($H_{1a}$, $H_{1b}$, $H_{2}$, $H_{3}$, $H_{4}$, or $H_{5}$) that minimizes the absolute error in each case. The MAE column suggests that the best mode to reduce the hardness calculation error is to simultaneously consider the *Crystal System and Density* classification (CLA$_2$). This model exhibits the lowest MAE of 2.2 GPa with a standard deviation of 2.2 GPa. The second best combination is *Crystal System and Bandgap* (CLA$_1$) followed by *Bandgap and Density* (CLA$_3$).

### The classic calculator

Even though the combination of *Crystal System and Density* exhibits the best result, the data presented in Table 4 reveals no statistical significant difference among the three combined methods (CLA$_1$, CLA$_2$ and CLA$_3$). Based on the latter observation, *The Classic Calculator* was developed as a selection model considering simple properties of a solid like crystal system, bandgap, and density.

**Table 5:** *The Classic Calculator* considering crystal system and bandgap simultaneously (CLA$_1$). Bandgap ($\Delta E$) was calculated theoretically. Materials are classified as insulators ($\Delta E > 2 eV$), semiconductors ($\Delta E < 2 eV$) and metals ($\Delta E =0$).

	Cubic	Hexagonal	Monoclinic	Orthorhombic	Tetragonal	Triclinic	Trigonal
Insulator	$H_{2}$	$H_{1b}$	$H_{2}$	$H_{2}$	$H_{4}$	$H_{5}$	$H_{2}$
Semiconductor	$H_{5}$	$H_{1a}$	$H_{4}$	$H_{2}$	$H_{1a}$	$H_{5}$	$H_{2}$
Metal	$H_{1a}$	$H_{4}$	$H_{4}$	$H_{4}$	$H_{4}$	$H_{4}$	$H_{2}$

**Figure 2:**
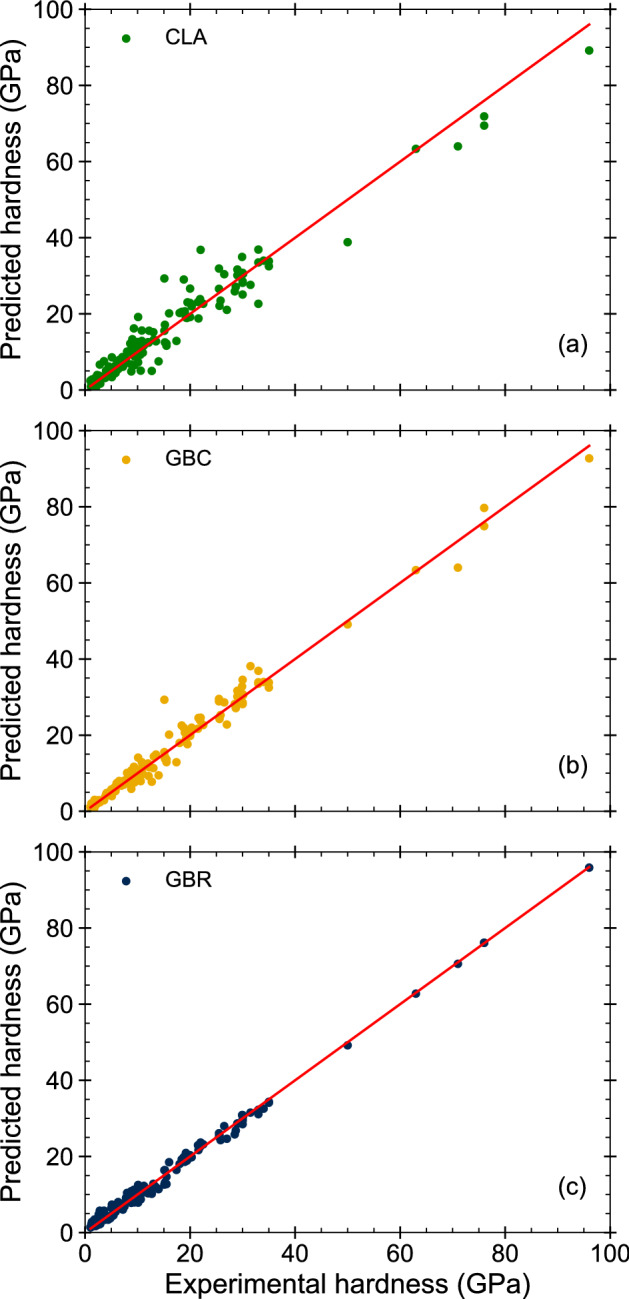
Comparison of the experimental Vickers hardness with the predicted values using: (**a**) *The Classic Calculator* as presented in Table 5 (CLA$_1$), (**b**) *The Machine Learning Calculator* using GBC and (**c**) GBR.

Table 5 summarizes the results considering the crystal system and the bandgap simultaneously. This table presents the relation that minimizes the error in the hardness calculation based on these two criteria. Figure 2a compares the experimental with the theoretical data calculated using this method. Most data points lie close to the red line, indicating that the calculated values greatly resemble the experimental data. The coefficient of determination ($R^2 = 0.95$) between the observed and estimated values also shows a strong correlation validating the model.

**Table 6:** *The Classic Calculator* considering crystal system and density simultaneously (CLA$_2$). Materials are classified by density ($\rho$) as low ($\rho <4$ g/cm$^3$), medium (4 g/cm$^3 \le \rho \le$ 9 g/cm$^3$) and high density ($\rho>$ 9 g/cm$^3$).

	Cubic	Hexagonal	Monoclinic	Orthorhombic	Tetragonal	Triclinic	Trigonal
Low	$H_{5}$	$H_{1b}$	$H_{1b}$	$H_{2}$	$H_{2}$	$H_{5}$	$H_{2}$
Medium	$H_{1a}$	$H_{4}$	$H_{4}$	$H_{4}$	$H_{4}$	$H_{4}$	$H_{4}$
High	$H_{1a}$	$H_{4}$		$H_{1a}$	$H_{3}$		

Similarly, Table 6 presents the results of simultaneously considering the crystal system and density, and Table 7 the bandgap and density. Any of the three different approaches of the *The Classic Calculator* can be used to select a proper relation for calculating hardness depending on the available information.

**Table 7:** *The Classic Calculator* considering bandgap and density simultaneously (CLA$_3$). Materials are classified by bandgap ($\Delta E$) as insulators ($\Delta E > 2$ eV), semiconductors ($\Delta E < 2$ eV) and metals ($\Delta E =0$); and by density ($\rho$) as low ($\rho <4$ g/cm$^3$), medium (4 g/cm$^3 \le \rho \le$ 9 g/cm$^3$) and high density ($\rho>$ 9 g/cm$^3$).

	Low	Medium	High
Insulator	$H_{2}$	$H_{2}$	
Semiconductor	$H_{5}$	$H_{4}$	$H_{3}$
Metal	$H_{2}$	$H_{4}$	$H_{4}$

For example, diamond is a low-density ($\rho = 3.5$ $g/cm^3$) insulator ($\Delta E = 4.3$ eV) with a cubic crystal system ($\rho$ and $\Delta E$ correspond to theoretical values extracted from the Materials Project’s database). Table 5 displays the classic calculator considering crystal system and the bandgap simultaneously (CLA$_1$). In the case of diamond, the latter suggests using relation $H_{2}$ (89.3 GPa) to estimate the hardness of diamond. Table 6 is the classic calculator considering crystal system and density simultaneously (CLA$_2$). For diamond, CLA$_2$ suggests using relation $H_{5}$ (93.0 GPa) to compute hardness. Table 7 shows the classic calculator built upon bandgap and density (CLA$_3$). In the case of diamond CLA$_3$ recommends using relation $H_{2}$ (89.3 GPa) for hardness. As observed the three classic models display very similar results, but one can be more accurate than the other. Given the experimental Vickers hardness of diamond is 96 GPa, CLA$_2$ exhibits the best prediction, which agrees with the results presented in Table 4. However, any of the classic models may be used to estimate hardness depending on the available information.

### The machine learning calculator

**Table 8:** Machine learning for hardness prediction. Out-of-sample accuracy and Jaccard index for different machine learning algorithms. The MAE (GPa) and standard deviation $\sigma$ (GPa) consider the entire dataset. Classification algorithms (C) target the best calculation relation, and the regression algorithms (R) the hardness value directly.

Algorithm	Type	Accuracy	Jaccard	MAE	$\sigma$
KNN	C	21%	12%	2.3	2.9
DT	C	31%	18%	2.9	3.7
LR	C	14%	7%	3.5	4.4
SVM	C	14%	7%	2.9	3.2
RF	C	14%	7%	3.3	4.3
ADA	C	7%	4%	3.9	4.0
GBC	C	31%	18%	1.4	1.9
GBR	R	n/a	n/a	1.3	1.9

Table 8 displays the performance of different supervised machine learning techniques when trying to solve the hardness problem. The results for seven different classification methods and one regression algorithm are shown and compared to each other.

#### Classification

The classification algorithms target the best calculation relation in each case. As observed in Table 8, GBC (31%) and DT (31%) have the highest accuracy, followed by KNN (21%). The Jaccard index reflects, almost identically, the same behavior. At first glance, 31% accuracy may suggest a low performance. However, this not necessarily means the classifier did a poor job because some materials can work successfully with two, three, or four hardness relations. Therefore, to keep a more balanced measure of the performance of the different classifiers, we have selected the best by minimizing the MAE. GBC presented the lowest MAE (1.4 GPa), followed by KNN (2.3 GPa), DT (2.9 GPa) and SVM (2.9 GPa). Also, GBC (1.9 GPa) exhibited the lowest standard deviation, followed by KNN (2.9 GPa) and SVM (3.2 GPa). Based on the latter results, it is indisputable that GBC is the best classifier, given its higher accuracy and low MAE.

GBC is a very sophisticated technique, so it is not surprising that it outperforms KNN or DT. However, it is remarkable to observe that even though KNN has a lower accuracy, its MAE is smaller than DT. This confirms the fact that materials with similar mechanical properties will work adequately with the same relation to estimate hardness ($H_{1a}$, $H_{1b}$, $H_{2}$, $H_{3}$, $H_{4}$, or $H_{5}$). On the other hand, DT had the same accuracy as GBC, but its MAE is very high, implying that for the unsuccessful samples the algorithm had a poor performance.

Figure 2b shows the experimental and predicted values of hardness using GBC. As observed, there is a clear linear trend corroborated by the coefficient of determination ($R^2 = 0.98$). Also, the dispersion of the data points in Fig. 2b is less than the one observed in Fig. 2a, suggesting that the GBC provides a better model for future forecasts than *The Classic Calculator*.

#### Regression

The results in the previous section show that the Gradient Boosting Classifier (GBC) is the best algorithm to select the hardness calculation relation given the properties of a solid. Gradient boosting is a robust algorithm used for regression or classification tasks. Given that the classifier did such an outstanding job, the Gradient Boosting Regressor (GBR) was implemented to predict the value of hardness directly in this study. As observed in Table 8, the performance of the regressor is better than the classifier. While the regressor displays a MAE of 1.3 GPa, the classifier shows 1.4 GPa, a small difference of 0.1 GPa that favors the regressor over the classifier. Additionally, the standard deviation of the regressor and the classifier have the same value, suggesting an overall better prediction by the regressor.

Comparing the MAE of GBR (1.3 GPa) with the best possible result (1.0 GPa) shown in Table 4, it is clear that the GBR works effectively predicting the value of hardness, followed by the GBC (1.4 GPa) and KNN (2.3 GPa). Also, GBR (1.9 GPa) and GBC (1.9 GPa) display the lowest standard deviation among all the ML techniques explored in this work, followed by KNN (2.9 GPa) and SVM (3.2 GPa). The standard deviations of GBR and GBC are only 0.7 GPa above the best possible result (1.2 GPa), a small value compared to the results exhibited by other methods. The latter results demonstrate that GBR has the best performance among all the ML algorithms evaluated in this work. Consequently, GBC holds second place, followed by KNN.

In the case of diamond, the classification algorithms KNN, DT, LR, SVM, RF, and GBC predicted the best relation is $H_{5}$ (93.0 GPa), while ADA inclined towards $H_{2}$ (89.3 GPa). On the other hand, the regressor GBR directly predicts a value of 95.9 GPa.

Figure 2c displays the experimental and predicted values of hardness using GBR. As observed most of the data points lie very close to the red line, minimizing the dispersion of the data. The coefficient of determination in this case ($R^2 = 0.99$) is very close to 1.0, indicating that the statistical model predicts hardness successfully. In Fig. 2c we can observe that GBR manages to correct some data points that were not predicted correctly neither by CLA or GBC. Given these observations, we recommend GBR as the most reliable method for predicting hardness, among all the different techniques proposed in this study.

### Prediction of hard and superhard materials

**Figure 3:**
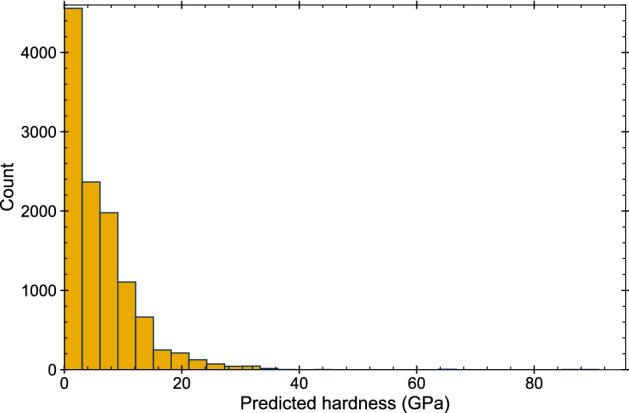
Histogram of the hardness values estimated using *The hardness calculator* for the Materials Project’s database^15^.

**Table 9:** Prediction of hard and superhard materials using *The Hardness Calculator*. Materials Project’s database identification number, chemical formula, crystal system (CS), bandgap ($\Delta E$ in eV), density ($\rho$ in $g/cm^3$), bulk modulus (*B* in GPa), shear modulus (*G* in GPa), Young’s modulus (*Y* in GPa) and Poisson’s ratio ($\nu$) are shown. Vickers hardness (in GPa) was calculated using *The Classic Calculator* (H$_{CLA_1}$) according to Table 5, and the *The Machine Learning Calculator* using the GBC (H$_{GBC}$) and the GBR (H$_{GBR}$). The comments specify whether the material was predicted to be hard by this work, by this work and other authors or if the hardness has been previously measured experimentally.

Material ID	Formula	CS	$\Delta E$	$\rho$	*B*	*G*	*Y*	$\nu$	H$_{CLA_1}$	H$_{GBC}$	H$_{GBR}$	Comment
mp-2653	BN	Hexag	5.4	3.5	373	383	856	0.12	52 ($H_{1b}$)	64 ($H_{5}$)	68	H$_{exp}$ = 46^a^
mp-1569	${\text {Be}_2}$C	Cubic	1.4	2.5	201	231	501	0.09	54 ($H_{5}$)	54 ($H_{5}$)	44	H$_{exp}$ > 34^b^
mp-2075	${\text {Si}_{3}}{\text {N}_4}$	Cubic	3.3	3.9	294	249	582	0.17	41 ($H_{2}$)	28 ($H_{4}$)	27	H$_{exp}$ = 35^c^
mp-1491	${\text {VB}_2}$	Hexag	0	5.1	286	241	565	0.17	27 ($H_{4}$)	27 ($H_{4}$)	27	H$_{exp}$ = 27.5^d^
mp-1994	${\text {HfB}_2}$	Hexag	0	11.1	251	242	550	0.13	27 ($H_{4}$)	33 ($H_{1b}$)	30	H$_{exp}$ = 31.5^e^
mp-2852	${\text {C}_3}{\text {N}_4}$	Cubic	3.0	3.8	416	380	874	0.15	64 ($H_{2}$)	64 ($H_{2}$)	69	TW & others^f^
mp-1008523	${\text {BC}_2}N$	Tetra	1.6	3.3	347	397	862	0.09	58 ($H_{1a}$)	74 ($H_{5}$)	72	TW & others^g^
mp-1009818	${\text {CN}_2}$	Tetra	0.2	3.6	404	288	697	0.21	42 ($H_{1a}$)	48 ($H_{2}$)	29	TW & others^h^
mp-15703	${\text {BeCN}_2}$	Tetra	3.9	3.3	316	292	669	0.15	32 ($H_{4}$)	32 ($H_{4}$)	32	TW & others^i^
mp-1008527	${\text {B}_2}\text {CN}$	Tetra	0	3.1	324	261	617	0.18	29 ($H_{4}$)	29 ($H_{4}$)	31	TW & others^j^
mp-1019055	${\text {ReN}_2}$	Tetra	0	13.8	380	254	622	0.23	28 ($H_{4}$)	37 ($H_{1a}$)	31	TW & others^k^
mp-867212	${\text {TcOs}_3}$	Hexag	0	19.3	378	244	602	0.23	27 ($H_{4}$)	36 ($H_{1a}$)	31	TW & others^l^
mp-1018050	CrC	Hexag	0	6.4	342	244	591	0.21	27 ($H_{4}$)	27 ($H_{4}$)	28	TW & others ^m^
mp-1019317	${\text {TcB}_2}$	Hexag	0	7.3	283	244	568	0.17	27 ($H_{4}$)	27 ($H_{4}$)	27	TW & others^n^
mp-1009735	ReC	Hexag	0	16.2	412	233	589	0.26	26 ($H_{4}$)	38 ($H_{2}$)	33	TW & others^o^
mp-571653	${\text {C}_3}{\text {N}_4}$	Cubic	2.8	3.7	394	382	866	0.13	65 ($H_{2}$)	42 ($H_{4}$)	71	This work
mp-1985	${\text {C}_3}{\text {N}_4}$	Hexag	3.3	3.5	409	313	747	0.20	45 ($H_{1b}$)	52 ($H_{2}$)	29	This work
mp-999498	${\text {N}_2}$	Cubic	4.0	3.4	276	241	561	0.16	40 ($H_{2}$)	27 ($H_{4}$)	30	This work
mp-1019740	${\text {GaB}_3}{\text {N}_4}$	Cubic	3.7	4.5	329	229	558	0.22	38 ($H_{2}$)	25 ($H_{4}$)	28	This work
mp-1008630	WC	Cubic	0	15.9	358	231	571	0.23	34 ($H_{1a}$)	34 ($H_{1a}$)	30	This work
mp-1002105	VN	Cubic	0	6.5	264	231	536	0.16	34 ($H_{1a}$)	26 ($H_{4}$)	29	This work
mp-999549	${\text {WN}_2}$	Hexag	1.5	12.1	353	226	559	0.24	33 ($H_{1a}$)	33 ($H_{1a}$)	30	This work
mp-1330	AlN	Cubic	4.6	4.0	255	217	508	0.17	36 ($H_{2}$)	31 ($H_{1b}$)	28	This work
mp-1010	${\text {MnB}_4}$	ortho	0	4.4	261	240	551	0.15	27 ($H_{4}$)	27 ($H_{4}$)	31	This work
mp-2305	MoC	Hexag	0	8.5	350	240	586	0.22	27 ($H_{4}$)	27 ($H_{4}$)	30	This work
mp-644751	BN	Ortho	5.7	3.0	303	215	521	0.21	35 ($H_{2}$)	24 ($H_{4}$)	26	This work
mp-1082	${\text {VIr}_3}$	Cubic	0	18.4	320	215	527	0.23	32 ($H_{1a}$)	32 ($H_{1a}$)	29	This work
mp-265	${\text {TaIr}_3}$	Cubic	0	20.8	325	213	524	0.23	31 ($H_{1a}$)	31 ($H_{1a}$)	29	This work
mp-1009471	NbN	Cubic	0	8.5	316	210	517	0.23	31 ($H_{1a}$)	31 ($H_{1a}$)	29	This work
mp-1459	TaN	Hexag	0	14.8	338	238	578	0.21	26 ($H_{4}$)	26 ($H_{4}$)	28	This work
mp-12083	${\text {CrIr}_3}$	Cubic	0	18.6	307	214	521	0.22	32 ($H_{1a}$)	32 ($H_{1a}$)	27	This work

^a^Ref.^26^; ^b^Ref.^27^; ^c^Ref.^28^; ^d^Ref.^29^; ^e^Ref.^30^; ^f^Ref.^31^; ^g^ Ref.^32^; ^h^Ref.^33^; ^i^Ref.^34^; ^j^Ref.^35^; ^k^Ref.^36^; ^l^Ref.^37^; ^m^ Ref.^38^; ^n^Ref.^39^; ^o^Ref.^40^.

The Materials Project’s database was explored for compounds with the computed elastic tensor. Approximately 12,000 materials meet the criteria. The mechanical properties (B, G, Y, $\nu$) were calculated for each one of them using the MechElastic package^16^. The materials were further classified (by crystal system, density, and bandgap) using the theoretical data provided by the Materials Project. The hardness was estimated using the *Classic* and the *Machine Learning Calculator*. Figure 3 presents the histogram for the predicted values of hardness for the Materials Project’s database. As observed, most materials (78.2%) exhibit hardness values below 10 GPa, and 18.2% have hardness values between 10 and 19 GPa. Hard materials, with values between 20 and 39 GPa, represent only 3.5% of the database. Superhard materials, those that exhibit Vickers hardness above 40 GPa^41^, are very scarce; only 0.2% of the materials in the database are candidates to be superhard.

Table 9 presents some of the materials predicted to be hard and superhard using *The Hardness Calculator*. From this list, we found that five materials have experimental hardness measurements, ten have been predicted to be hard by other authors, and the remaining sixteen are predicted to be hard within this work.

The compounds BN, ${\text {Be}_2}\text {C}$, ${\text {Si}_3}{\text {N}_4}$, ${\text {VB}_2}$ and ${\text {HfB}_2}$ have been previously synthesized and were predicted to be superhard at least by one of the methods presented in Table 9. Even though, in general, the experimental values are slightly below the predictions, BN is experimentally superhard, and the rest of the materials are hard, corroborating the goodness of the methods implemented in *The Hardness Calculator*.

In agreement with our predictions, other theoretical studies have suggested that ${\text {C}_3}{\text {N}_4}$, ${\text {BC}_2}\text {N}$ and ${\text {CN}_2}$ are excellent candidates to be superhard materials. From first-principles calculations, Teter et al. predicted a cubic form of ${\text {C}_3}{\text {N}_4}$ with a zero-pressure bulk modulus exceeding that of diamond. The authors suggested that this phase could potentially be synthesized for use as a superhard material^31^. Also, Hong Sun et al. studied different cubic ${\text {BC}_2}\text {N}$ structures from ab initio methods^32^. The authors stated that the two hardest c-${\text {BC}_2}\text {N}$ structures have bulk and shear moduli comparable to or slightly higher than c-BN, suggesting these compounds are superhard. They also believe these structures are similar to c-${\text {BC}_2}\text {N}$ synthesized by Knittle et al.^42^. However, the experimental hardness of this compound is still unknown. Finally, Quan Li et al. predicted the body-centered tetragonal structure of $\text {CN}_2$ from first principles^33^. The authors simulated a hardness of 77 GPa for this compound, indicating that it has excellent incompressible and superhard properties. Similarly, other authors have suggested that ${\text {BeCN}_2}$, ${\text {B}_2}\text {CN}$, ${\text {ReN}_2}$, ${\text {TcOs}_3}$, CrC, ${\text {TcB}_2}$, and ReC are good candidates for hard materials. All these observations suggest that the methods implemented in *The hardness calculator* are coherent with the findings in previous studies.

To our knowledge, the remaining sixteen materials proposed to be hard in this work have not yet been studied for hardness. We hope this work motivates the experimental study of these compounds.

### Website

*The Hardness Calculator* is a standalone online application created for simple analysis of hardness (available at https://www.hardnesscalculator.com). It is a user-friendly interface that requires mechanical properties as an input to compute the hardness of a material. The program displays the hardness values calculated by *The Machine Learning Calculator* ($H_{GBC}$ and $H_{GBR}$) as well as all the other values of hardness estimated by the six different relations described in Sect. 2.1 ($H_{1a}$, $H_{1b}$, $H_{2}$, $H_{3}$, $H_{4}$, and $H_{5}$). If the user provides the crystal system, density and/or bandgap, the program will also indicate the preferred relation to estimate hardness according to *The Classic Calculator*.

## Conclusions

In this study, we have discussed several methodologies to compute hardness using the mechanical properties of a solid (bulk modulus, shear modulus, Young’s modulus, and Poisson’s ratio) as input variables. We have approached the hardness estimation problem from two different perspectives.

In the first approach, we investigated the correlation between different hardness relations ($H_{1a}$, $H_{1b}$, $H_{2}$, $H_{3}$, $H_{4}$, and $H_{5}$) and some physical properties of solids, such as crystal system, bandgap, and density. From this first part, we developed *The Classic Calculator*, which is a selection model based on the simple properties of a solid. The best results were observed considering two properties simultaneously: *Crystal System + Bandgap*, *Crystal System + Density*, or *Bandgap + Density*. The MAE (standard deviation) in the hardness calculation for each one of these methods is 2.3 GPa (2.7 GPa), 2.2 GPa (2.2 GPa), and 2.5 GPa (2.9 GPa), respectively. Even thou the combination of *Crystal System + Density* exhibits the better performance among the three approaches, there is no significant statistical difference between these methods; any of them can be used to select the proper relation to calculate hardness depending on the available information.

The second approach is based on Machine Learning and is referred to as *The Machine Learning Calculator*. We proposed two models to compute hardness using ML: a classifier (GBC) and a regressor (GBR). The classifier targets the best relation to calculate the crystal hardness using the mechanical properties of a solid as input variables. On the other hand, the regressor directly predicts the hardness value using the same input variables as the classifier. GBC and GBR display a MAE (standard deviation) of 1.4 GPa (1.9 GPa) and 1.3 GPa (1.9 GPa), respectively. GBR displays the best performance among all the different techniques studied in this work.

*The Hardness Calculator*, composed of classic and ML schemes, was used to search for hard and superhard materials within the Materials Project’s database. This exploration demonstrated that *The Hardness Calculator* shows great predictive power as our results match other experimental or theoretical studies. As a result, sixteen materials were proposed as new hard or super hard candidates by this work.

*The Hardness Calculator* is available as a free access online application for users to discriminate between the different results at https://www.hardnesscalculator.com.

## Supplementary Information

Supplementary Information.

## Data Availability

The authors declare that all data that support the findings of this study are included in the paper and/or its supplementary information files.

## Code availability

The codes comparing the performance of the different machine learning algorithms as well as the classifications by crystal system, bandgap and density available at https://github.com/vdovale29/Hardness-Calculator. The code performing the calculations for *The Hardness Calculator* are available at https://github.com/vdovale29/Hardness-Calculator.
