# Supplementary material for: Vickers hardness prediction from machine learning methods
Source: Sci Rep. 2022 Dec 28;12:22475. doi: 10.1038/s41598-022-26729-3 (PMC9797558; doi:10.1038/s41598-022-26729-3)
Supplement: Supplementary file 1 — Supplementary Information. [file 41598_2022_26729_MOESM1_ESM.pdf]

The Hardness Calculator (Supplemental Information)

Viviana Dovale-Farelo[1,*], Pedram Tavadze[1], Logan Lang[1], A. Bautista-Hernandez[2], and Aldo H. Romero[1]

[1]Department of Physics, West Virginia University, Morgantown, WV 26506.
[2]Facultad de Ingeniería, Benemérita Universidad Autónoma de Puebla, Edificio ING2, Ciudad Universitaria, CP. 72570 Puebla, México.
[*]Corresponding author: vd0020@mix.wvu.edu

July 2022

List of materials used in this study

The following table specifies the list of materials used in the present study. In the heading: the Materials Project's identification number, chemical formula, space group (SPG), crystal system (SC), bandgap (ΔE in eV) and density (ρ in g/cm^3). The bandgap and density are theoretical values extracted from the Materials Project's database [1]. The bulk modulus (B in GPa), shear modulus (G in GPa), Young's modulus (Y in GPa) and Poisson's ratio (ν) were calculated from the elastic tensor. The asterisk (*) next to the Material ID specifies that the elastic tensor was calculated in this work, otherwise it was extracted directly from the Materials Project's database. Lastly, the experimental Vickers hardness (H_{exp} in GPa) and the reference for H_{exp} are shown.

No.	Material ID	Formula	SPG	CS	ΔE	ρ	B	G	Y	ν	H_{exp}	Ref.
1	mp-66	Diamond	227	cubic	4.3	3.5	435	521	1117	0.07	96.0	[2]
2	mp-1639	BN	216	cubic	4.6	3.5	408	374	860	0.15	63.0	[3]
3	mp-8062	SiC	216	cubic	1.6	3.2	211	187	433	0.16	34.0	[2]
4	mp-1479	BP	216	cubic	1.5	3.0	160	162	364	0.12	33.0	[3]
5	mp-9258	SiO_2	205	cubic	4.5	4.4	320	206	508	0.24	33.0	[3]
6	mp-1086	TaC	225	cubic	0.0	14.2	324	215	527	0.23	29.0	[3]
7	mp-1282	VC	225	cubic	0.0	5.8	307	204	501	0.23	29.0	[2]
8	mp-2030	RuS_2	205	cubic	0.7	6.1	131	121	278	0.15	28.7	[4]
9	mp-631	TiC	225	cubic	0.0	4.9	252	176	428	0.22	28.5	[3]
10	mp-1352	ZrN	225	cubic	0.0	7.1	247	143	359	0.26	27.0	[3]
11	mp-2795	ZrC	225	cubic	0.0	6.5	221	159	385	0.21	25.8	[3]
12	mp-21075	HfC	225	cubic	0.0	12.6	236	180	431	0.20	25.5	[3]
13	mp-1008785	RuO_2	225	cubic	0.7	7.7	282	185	455	0.23	20.0	[2]
14	mp-1580	NbN	225	cubic	0.0	8.0	305	130	340	0.31	20.0	[3]
15	mp-492	TiN	225	cubic	0.0	5.3	259	181	440	0.22	20.0	[3]
16	mp-2828	HfN	225	cubic	0.0	13.7	266	156	392	0.25	19.5	[3]
17	mp-90	Cr	229	cubic	0.0	7.3	259	128	330	0.29	19.4	[4]
18	mp-910	NbC	225	cubic	0.0	7.6	300	197	484	0.23	18.8	[3]
19	mp-20905	OsS_2	205	cubic	0.0	9.3	193	139	336	0.21	18.4	[4]
20	mp-226	FeS_2	205	cubic	0.5	5.1	146	135	310	0.15	15.1	[4]
21	mp-1565	ZrO_2	225	cubic	3.3	6.0	235	102	267	0.31	13.0	[3]
22	mp-149	Si	227	cubic	0.9	2.3	83	61	148	0.20	12.0	[2]
23	mp-2513	As_2Pt	205	cubic	0.3	10.3	137	83	207	0.25	11.2	[4]

No.	Material ID	Formula	SPG	CS	ΔE	ρ	B	G	Y	ν	H_{exp}	Ref.
24	mp-2070	CoS_2	205	cubic	0.0	4.9	124	73	183	0.25	10.7	[4]
25	mp-2490	GaP	216	cubic	1.7	4.0	76	52	126	0.22	9.5	[2]
26	mp-1550	AlP	216	cubic	1.8	2.3	85	47	119	0.27	9.4	[2]
27	mp-32	Ge	227	cubic	0.0	5.0	59	46	109	0.19	8.8	[3]
28	mp-452	$CoAs_3$	204	cubic	0.0	6.7	116	83	201	0.21	8.6	[4]
29	mp-2534	GaAs	216	cubic	0.2	5.1	60	41	100	0.22	7.5	[2]
30	mp-2652	Y_2O_3	206	cubic	4.1	4.9	138	62	161	0.31	7.5	[2]
31	mp-562	Sb_2Pt	205	cubic	0.0	10.4	104	54	139	0.28	7.5	[4]
32	mp-3830	NiAsS	198	cubic	0.0	6.0	109	59	149	0.27	7.1	[4]
33	mp-3679	NiSbS	198	cubic	0.0	6.8	96	51	129	0.27	6.1	[4]
34	mp-2210	ZrCu	221	cubic	0.0	7.3	117	30	84	0.38	5.8	[2]
35	mp-20901	$NiSe_2$	205	cubic	0.0	6.9	77	38	98	0.29	5.4	[4]
36	mp-20351	InP	216	cubic	0.5	4.6	60	31	80	0.28	5.4	[2]
37	mp-22864	Bi_2Pt	205	cubic	0.0	12.8	82	23	63	0.37	5.1	[4]
38	mp-2172	AlAs	216	cubic	1.7	3.6	70	39	99	0.26	5.0	[2]
39	mp-22682	In_2Pt	225	cubic	0.0	10.4	95	30	80	0.36	4.9	[4]
40	mp-1068	CuS_2	205	cubic	0.0	4.3	78	34	90	0.31	4.8	[4]
41	mp-4368	SbPdSe	198	cubic	0.0	7.7	85	31	83	0.34	4.7	[4]
42	mp-1156	GaSb	216	cubic	0.4	5.3	45	30	74	0.23	4.5	[2]
43	mp-21765	$Pd_{17}Se_{15}$	221	cubic	0.0	7.9	97	26	72	0.38	4.1	[4]
44	mp-2624	AlSb	216	cubic	1.3	4.1	49	30	74	0.25	4.0	[2]
45	mp-20305	InAs	216	cubic	0.3	5.3	49	25	65	0.28	3.8	[2]
46	mp-718	$SnPd_3$	221	cubic	0.0	11.0	146	51	138	0.34	3.6	[4]
47	mp-738	Sb_2Au	205	cubic	0.0	9.3	71	23	63	0.35	2.9	[4]
48	mp-20849	Pd_3Pb	221	cubic	0.0	12.5	131	45	122	0.35	2.8	[4]
49	mp-20012	InSb	216	cubic	0.0	5.4	35	19	48	0.27	2.2	[2]
50	mp-559200	$Cd(InS_2)_2$	227	cubic	1.5	4.7	63	22	60	0.34	2.1	[4]
51	mp-570113	$BiAu_2$	227	cubic	0.0	15.1	102	21	58	0.41	2.1	[4]
52	mp-361	Cu_2O	224	cubic	0.5	6.0	111	9	25	0.46	2.0	[4]
53	mp-10695	ZnS	216	cubic	2.4	4.0	69	33	86	0.29	1.8	[2]
54	mp-1190	ZnSe	216	cubic	1.6	5.1	59	27	71	0.30	1.4	[2]
55	mp-2176	ZnTe	216	cubic	1.1	5.4	46	23	58	0.29	1.0	[2]
56	mp-1145	TiB_2	191	hexag	0.0	4.5	253	253	569	0.13	31.5	[3]
57	mp-1773	ReB_2	194	hexag	0.0	12.5	338	270	639	0.18	30.1	[2]
58	mp-1472	ZrB_2	191	hexag	0.0	6.0	238	226	515	0.14	30.0	[3]
59	mp-1894	WC	187	hexag	0.0	15.4	385	279	674	0.21	30.0	[2]
60	mp-1108	TaB_2	191	hexag	0.0	12.1	302	199	490	0.23	25.6	[3]
61	mp-988	Si_3N_4	176	hexag	4.3	3.1	234	120	309	0.28	19.0	[3]
62	mp-661	AlN	186	hexag	4.1	3.2	195	122	302	0.24	18.0	[3]
63	mp-763	MgB_2	191	hexag	0.0	2.6	148	117	278	0.19	17.4	[3]
64	mp-374	CrB_2	191	hexag	0.0	5.3	252	183	442	0.21	16.0	[5]
65	mp-960	$a{-}B_2Mo$	191	hexag	0.0	7.3	299	153	392	0.28	15.2	[6]
66	mp-804	GaN	186	hexag	1.7	5.9	172	105	262	0.25	15.1	[2]
67	mp-778	Fe_2P	189	hexag	0.0	7.1	216	89	234	0.32	11.0	[4]
68	mp-1111	Mn_5Si_3	193	hexag	0.0	6.0	148	46	125	0.36	10.6	[4]
69	mp-22205	InN	186	hexag	0.5	6.6	105	66	164	0.24	9.0	[2]
70	mp-2133	ZnO	186	hexag	0.7	5.4	130	41	112	0.36	7.2	[3]
71	mp-504974	$Fe_2Mo_3O_8$	186	hexag	0.3	5.4	141	42	115	0.36	6.0	[4]
72	mp-1070	CdSe	186	hexag	0.6	5.3	44	15	41	0.35	2.1	[4]
73	mp-504	CuS	194	hexag	0.0	4.6	80	25	67	0.36	1.3	[4]
74	mp-561551*	$Y_2Si_2O_7$	11	monoc	5.2	3.9	105	59	149	0.26	14.0	[4]
75	mp-759686*	$BeAlSiHO_5$	14	monoc	5.7	3.0	147	91	227	0.24	13.1	[4]
76	mp-561511	FeAsS	14	monoc	0.7	6.2	143	117	276	0.18	10.8	[4]

No.	Material ID	Formula	SPG	CS	ΔE	ρ	B	G	Y	ν	H_{exp}	Ref.
77	mp-2715	$CoAs_2$	14	monoc	0.2	7.4	132	89	218	0.22	8.0	[4]
78	mp-558553*	$Sr_4Ti_5(Si_2O_{11})_2$	12	monoc	2.0	4.0	127	57	149	0.30	7.1	[4]
79	mp-1191211*	$TiVO_4$	14	monoc	1.3	4.1	146	73	188	0.29	6.5	[4]
80	mp-704645	CuO	15	monoc	0.0	5.9	146	25	72	0.42	2.5	[4]
81	mp-10519	Cu_2SnS_3	9	monoc	0.0	4.6	66	27	72	0.32	1.8	[4]
82	mp-30148	BC_2N	17	ortho	2.1	3.4	361	423	912	0.08	76.0	[2]
83	mp-629458	BC_2N	25	ortho	1.8	3.4	362	409	891	0.09	76.0	[3]
84	mp-1193675*	B	58	ortho	1.5	2.6	223	236	523	0.11	50.0	[2]
85	mp-10142	Ta_3B_4	71	ortho	0.0	13.3	296	208	506	0.22	33.0	[7]
86	mp-27710	CrB_4	71	ortho	0.0	4.3	277	258	590	0.15	30.0	[5]
87	mp-568803	$Mg(B_6C)_2$	74	ortho	2.3	2.7	229	214	489	0.14	29.9	[8]
88	mp-8204*	$LiAlB_{14}$	74	ortho	1.3	2.5	194	188	427	0.13	26.5	[8]
89	mp-34763*	$NaAlB_{14}$	74	ortho	1.7	2.7	193	197	440	0.12	25.5	[8]
90	mp-889	Cr_3B_4	71	ortho	0.0	5.9	299	210	511	0.21	21.9	[7]
91	mp-2850	B_2Os	59	ortho	0.0	12.6	311	166	423	0.27	21.6	[3]
92	mp-973391*	$LiSiB_6$	64	ortho	1.7	2.3	163	141	328	0.17	20.3	[8]
93	mp-1077	B_2Ru	59	ortho	0.0	7.5	285	167	420	0.25	19.2	[3]
94	mp-4571	$CaZrO_3$	62	ortho	3.8	4.5	148	85	215	0.26	15.5	[4]
95	mp-9270	SbIrS	29	ortho	1.3	10.1	143	87	217	0.25	15.2	[4]
96	mp-4627	CoAsS	29	ortho	0.9	6.3	139	104	251	0.20	12.2	[4]
97	mp-766	As_2Ru	58	ortho	0.6	8.1	149	105	255	0.22	10.8	[4]
98	mp-1522	FeS_2	58	ortho	1.0	4.9	150	125	293	0.17	10.1	[4]
99	mp-5881	CoSbS	61	ortho	0.4	6.9	116	79	192	0.22	9.8	[4]
100	mp-427	FeAs	62	ortho	0.0	8.1	163	81	208	0.29	9.4	[4]
101	mp-760	$FeSe_2$	58	ortho	0.4	7.1	118	92	218	0.19	9.0	[4]
102	mp-553946	CoAsS	31	ortho	0.0	6.2	134	96	232	0.21	8.8	[4]
103	mp-4962	CoSbS	31	ortho	0.0	6.8	113	75	184	0.23	7.8	[4]
104	mp-22079	AsRh	62	ortho	0.0	9.2	156	61	161	0.33	7.4	[4]
105	mp-19814	$NiAs_2$	58	ortho	0.0	6.9	114	54	140	0.29	6.9	[4]
106	mp-5305	CuAsS	62	ortho	0.0	4.8	67	29	77	0.31	2.5	[4]
107	mp-649774	$CuSbPbS_3$	31	ortho	0.8	5.6	21	13	33	0.24	1.9	[4]
108	mp-2273	Ag_3Sb	25	ortho	0.0	9.1	65	17	47	0.38	1.7	[4]
109	mp-6947	SiO_2	136	tetra	5.5	4.1	272	204	489	0.20	22.5	[4]
110	mp-856	SnO_2	136	tetra	1.4	6.6	172	87	222	0.28	13.5	[4]
111	mp-558986*	$CaMn_6SiO_{12}$	142	tetra	0.7	4.4	204	84	222	0.32	12.1	[4]
112	mp-1205321*	PtS	84	tetra	0.2	10.7	145	49	133	0.35	10.1	[4]
113	mp-3224*	Mn_7SiO_{12}	142	tetra	0.4	4.6	207	85	225	0.32	10.0	[4]
114	mp-2657	TiO_2	136	tetra	1.8	4.1	209	110	280	0.28	9.3	[4]
115	mp-288	PtS	131	tetra	0.5	9.9	156	33	93	0.40	8.8	[4]
116	mp-390	TiO_2	141	tetra	2.1	3.8	179	55	149	0.36	6.6	[4]
117	mp-5058	$FeAgS_2$	122	tetra	0.0	4.7	58	15	41	0.38	2.9	[4]
118	mp-22736	$InCuS_2$	122	tetra	0.3	4.6	64	25	67	0.32	2.7	[4]
119	mp-22648	$FeCu_2SnS_4$	121	tetra	0.0	4.5	68	25	67	0.33	2.4	[4]
120	mp-1825	Cu_2Sb	129	tetra	0.0	8.4	92	35	92	0.33	2.3	[4]
121	mp-505244	$FeCuSe_2$	111	tetra	0.0	5.5	28	14	35	0.29	2.0	[4]
122	mp-3497	$FeCuS_2$	122	tetra	0.0	4.3	56	19	52	0.35	1.9	[4]
123	mp-22795	Pb_2Au	140	tetra	0.0	12.5	57	13	35	0.40	1.5	[4]
124	mp-6763*	$K_2Ti(Si_2O_5)_3$	2	tricl	3.3	2.7	72	39	99	0.27	5.5	[4]
125	mp-556753*	$V_2Cu_5(HO_3)_4$	2	tricl	0.0	4.2	50	27	68	0.27	4.4	[4]
126	mp-510589	$MnCuO_2$	2	tricl	0.2	5.2	112	41	111	0.34	2.6	[4]
127	mp-1204830*	$As_9Pb_5S_{18}$	2	tricl	0.0	4.9	13	6	16	0.29	1.8	[4]
128	mp-1196185*	$V_4Cu_9(ClO_9)_2$	2	tricl	0.0	4.1	39	24	60	0.25	1.7	[4]
129	mp-1018649	$c-BC_5$	156	trigo	0.0	3.3	406	378	866	0.14	71.0	[2]

No.	Material ID	Formula	SPG	CS	ΔE	ρ	B	G	Y	ν	H_{exp}	Ref.
130	mp-1346	B_6O	166	trigo	2.2	2.6	227	208	477	0.15	35.0	[3]
131	mp-160	B	166	trigo	1.5	2.5	211	200	456	0.14	35.0	[3]
132	mp-696746	B_4C	166	trigo	1.6	2.5	226	178	423	0.19	30.0	[3]
133	mp-2331	$b-B_2Mo$	166	trigo	0.0	7.0	295	225	537	0.20	22.0	[6]
134	mp-1143	Al_2O_3	167	trigo	6.0	3.9	232	147	364	0.24	21.5	[3]
135	mp-19502*	$BaTi_{18}Mn_3O_{38}$	148	trigo	0.0	4.4	129	82	203	0.24	15.5	[4]
136	mp-3285*	$Na_2Ta_4O_{11}$	167	trigo	3.5	7.5	154	45	122	0.37	12.7	[4]
137	mp-19770*	Fe_2O_3	167	trigo	0.0	5.1	259	69	189	0.38	10.5	[4]
138	mp-19306	Fe_3O_4	166	trigo	0.0	4.9	176	64	172	0.34	7.4	[4]
139	mp-19417	$TiFeO_3$	148	trigo	2.0	4.6	167	47	129	0.37	6.3	[4]
140	mp-14142	$TiZnO_3$	148	trigo	3.0	5.0	170	51	139	0.36	5.5	[4]
141	mp-19082	$TiMnO_3$	148	trigo	2.0	4.5	159	65	171	0.32	5.1	[4]
142	mp-11	As	166	trigo	0.0	5.5	41	28	68	0.22	1.2	[4]
143	mp-782	Te_2Pd	164	trigo	0.0	7.9	53	22	57	0.32	1.1	[4]

References

[1] Jain, A. *et al.* Commentary: The Materials Project: A materials genome approach to accelerating materials innovation. *APL Materials* **1**, 011002 (2013).

[2] Chen, X.-Q., Niu, H., Li, D. & Li, Y. Modeling hardness of polycrystalline materials and bulk metallic glasses. *Intermetallics* **19**, 1275–1281 (2011).

[3] Jiang, X., Zhao, J. & Jiang, X. Correlation between hardness and elastic moduli of the covalent crystals. *Computational materials science* **50**, 2287–2290 (2011).

[4] Ida Chau, J. R. mindat.org.

[5] Wang, S. *et al.* Crystal structures, elastic properties, and hardness of high-pressure synthesized CrB_2 and CrB_4. *Journal of Superhard Materials* **36**, 279–287 (2014).

[6] Tao, Q. *et al.* Enhanced Vickers hardness by quasi-3D boron network in MoB_2. *RSC advances* **3**, 18317–18322 (2013).

[7] Miao, N., Sa, B., Zhou, J. & Sun, Z. Theoretical investigation on the transition-metal borides with Ta_3B_4-type structure: A class of hard and refractory materials. *Computational materials science* **50**, 1559–1566 (2011).

[8] Jiang, X., Zhao, J., Wu, A., Bai, Y. & Jiang, X. Mechanical and electronic properties of B_12-based ternary crystals of orthorhombic phase. *Journal of Physics: Condensed Matter* **22**, 315503 (2010).
